# Governance and responses of health and surveillance systems to COVID-19 in BRICS countries: A scoping review protocol

**DOI:** 10.1371/journal.pone.0319572

**Published:** 2025-03-17

**Authors:** Adelyne Maria Mendes Pereira, Monique Azevedo Esperidião, Sílvia Karla Azevedo Vieira Andrade, João Felipe Marques Silva, Louise Celeste Rolim Silva, Adriano Silva, Isabela Barboza da Silva Tavares Amaral, Isabel Domingos Martinez dos Santos, Thalyta Cássia de Freitas Martins, Aline Degrave

**Affiliations:** 1 Department of Health Planning and Administration, Sergio Arouca National School of Public Health, Oswaldo Cruz Foundation, Rio de Janeiro, Brazil; 2 Institute of Collective Health, Federal University of Bahia, Salvador, Bahia, Brazil; 3 Department of Collective Health, Londrina State University, Londrina, Paraná, Brazil; 4 Postgraduate Program in Public Health, Sergio Arouca National School of Public Health, Oswaldo Cruz Foundation, Rio de Janeiro, Brazil; 5 Institute of Communication and Scientific and Technological Information on Health, Oswaldo Cruz Foundation, Rio de Janeiro, Brazil; 6 Nursing Institute, Federal University of Rio de Janeiro, Macaé, Rio de Janeiro, Brazil; 7 Institute of Collective Health, Fluminense Federal University, Niterói, Rio de Janeiro, Brazil; 8 Department of Nursing, Federal University of Alfenas, Alfenas, Minas Gerais, Brazil; 9 Department of Humanities and Social Sciences, Université Paris Cité, Paris, France; NingboTech University, CHINA

## Abstract

The capacity of countries to respond to COVID-19 has varied, worsening inequities. Structural factors (such as socio-economic inequalities and health system financing conditions) and political-institutional factors (such as the leadership and directionality of government action) have a major influence on the structure and outcomes of national responses. An effective COVID-19 response requires strong national governance, coordination strategies, and integration between health system actions and public health surveillance measures. This scoping review aims to synthesize the evidence on the governance and responses of the BRICS countries to the COVID-19 pandemic within a context of structural inequalities, identifying lessons and gaps in the current literature. This protocol was guided by the scoping review methodology, developed by the Joanna Briggs Institute, and used the PRISMA-P reporting guidelines. Searches will be carried out on the BVS Portal, Pubmed, Web of Science, Scopus, Embase and Dimensions databases. Inclusion criteria include secondary sources in the form of scientific publications, complemented by government and institutional documents published between 2020 and 2024. Exclusion criteria exclude publications that do not involve the selected countries, focus only on local or regional level without contemplating national-level elements, and/or address with COVID-19 solely from a clinical perspective. The scoping review process will select and organize retrieved data using the Rayyan software. Six independent reviewers will select the articles, working in three pairs. The results will be described, analyzed, and categorized through a descriptive synthesis, correlating them with the research objectives and questions.

**Registration:**

This protocol is registered on the Open Science Framework (OSF) (available at https://doi.org/10.17605/OSF.IO/SN5ZY) and Figshare (available at https://doi.org/10.6084/m9.figshare.25908340) platforms.

## 1. Introduction

The COVID-19 pandemic is widely recognized as a major global health issue [1]. It has affected countries in different ways, triggering social, economic and political crises, as well as highlighting the health vulnerability of many of them [2]. Comparative analysis of health systems has proved valuable in identifying unique characteristics, successes, failures and determinants across national experiences, systematizing lessons for dealing with highly complex health emergencies [3–6]. The literature on comparative health systems involving the BRICS countries is relatively recent, and few studies have focused on the experience of coping with the context of COVID-19 [7,8]. Previous analyses have presented the experiences of individual countries specifically, making it clear that there is a need for research that explores and systematizes the complexities of the political and contextual characteristics that affect the health system and its results in controlling the pandemic, especially regarding the effects of inequalities.

The capacity of countries to respond to COVID-19 has varied, influenced by their international position and by structural and political-institutional constraints in each country. Countries considered less developed and affected by conflict often have chronically underfunded health systems and large populations living in social vulnerability. In these countries and populations, the consequences of COVID-19 are more extensive and generate greater inequalities [9]. Given the syndemic nature of COVID-19 [10], an effective response requires coordinated strategies across multiple sectors, including physical distancing, public spaces regulation, individual and collective protection measures, health systems reorganization at all levels, economic measures, and social protection policies [11,12]. These strategies must consider territorial dynamics and populations of greater social vulnerability [13–15].

Health authorities and experts have emphasized the importance of preparing the health system to be able to conduct vaccination, testing, early diagnosis, case monitoring and assistance at the various levels of care — primary and hospital — in a timely and effective manner [11,12,16]. Similarly, public health surveillance measures relating to territorial, environmental and genomic surveillance, along with the timely flow of information between health establishments and management departments across different levels of care at various territorial scales, have been highlighted as necessary strategies for responding to COVID-19 [12,17]. On the one hand, health systems resilience depends on adequate funding, infrastructure, workforce, equipment, medicines, and supply conditions [18]. On the other hand, it is strengthened by learning from previous epidemics - such as Ebola, severe acute respiratory syndrome (SARS) and Middle East respiratory syndrome - which appears to contribute to preventing future outbreaks [19].

In addition to these aspects, the response to COVID-19 depends on the implementation of effective national governance and coordination mechanisms [20]. Governance and communication with society have been highlighted as central strategies for a successful response to COVID-19 [21,22]. In this sense, issues related to informational governance [23] are particularly relevant. In the context of the response to COVID-19, Pereira [24, p. 75] defines governance as a set of strategies and actions developed with the aim of promoting national coordination of public policies to deal with the crisis generated by the pandemic, involving the formulation of an agreed national plan, defining the role of each level of government; the definition of a structure for governance, monitoring and evaluation of this plan; a balance between decentralization and centralization of strategies and actions; strengthening political and institutional capacities at the different instances of government; increasing mechanisms for diplomacy and intergovernmental cooperation.

Going forward from this definition, this Scoping Review Protocol considers that the governance of a national response to COVID-19 involves national coordination strategies and intersectoral actions in at least three dimensions: 1) Political-institutional governance, considering the articulation between different sectors (Health, Social Security and Social Assistance, Labor, Economy, among others) and levels of government; 2) Operational governance, dealing with the relationship between public health departments, institutions and services in the health system; and 3) Communicational governance, involving actions to promote information exchange between the state and civil society, workers’ organizations and companies [5,24].

The relationship between pandemics and health inequalities is well recognized in the light of historical experiences and, in the case of COVID-19, has also been associated with the social determination of health-disease processes and the inequities already observed in the socio-epidemiological profile related to chronic diseases [25]. The unequal conditions of the productive structure, the health system, the national science and technology system and the capacity of production and provision of supplies, all affect the response to COVID-19. Conjunctural, political and institutional factors also become relevant, given the importance of national leadership and coordination of health and multisectoral actions. Different response capacities mean that different countries and populations are heterogeneously affected, deepening inequalities and inequities [26].

The BRICS (Brazil, Russia, India, China and South Africa) is a group of populous, economically relevant countries, marked by significant structural inequalities, with different health systems and which have gathered a significant number of COVID-19 cases and deaths. In 2024, the number of SARS-CoV-2 infections in the BRICS countries reached 210.11 million, representing more than 27 per cent of the global total [27]. These reasons justify their selection as the focus of this scoping protocol. A cross-sectional study using data on the COVID-19 pandemic found an association between new daily cases of the disease and socioeconomic and demographic factors, available resources and the political response to the pandemic in BRICS countries. Between February 2020 and April 2021, India had the highest total number of confirmed cases (18.76 million), followed by Brazil (14.45 million), Russia (4.81 million) and South Africa (1.58 million), while China had 0.10 cases/million. South Africa had the lowest rate of vaccine doses administered (0.18 million) among the countries in the group on 30 April 2021 [28].

The BRICS countries’ response to COVID-19 has highlighted some similarities and differences in the actions of governments and the response capacity of their health systems. These differences can influence the lethality of the disease and the pace of vaccination, for example. Some intra-BRICS cooperation measures during the pandemic period were important, most notably loans to China, India, South Africa and Brazil through the New Development Bank through the emergency assistance program [29]. However, the pandemic also revealed conflicts and disputes between some countries in the bloc, resulting in limitations associated with the ability to formulate policies and the stages of following previously agreed positions [30].

The systematization of the main similarities and differences between the BRICS countries and the analysis of the capacity of state action in the face of the pandemic cannot be separated from a discussion about their conditioning factors, examining the global geopolitical context and the international relations in which they operate, as well as political-institutional aspects involving the governments and the socio-historical configuration of social protection and health systems. Along these lines, it is also essential to consider the multiple dimensions of social inequality present in these countries, and their differences in tackling and reducing inequality and poverty. Reducing inequality is a challenge for the BRICS countries, given their growing influence in the global economy [31]. Deep inequalities in health persist both within and between the BRICS countries [32], as well as the nature of poverty and inequality amongst them [33].

This scoping review protocol aims to present a study that aspires to systematize the evidence in national and international scientific literature regarding governance and national responses of the BRICS countries to the COVID-19 pandemic in a context of structural inequalities. Key aspects include the characteristics of governance and the coordination of national responses; the strategies adopted by health systems, including the surveillance systems; intersectoral coordination; risk communication and the participation of general society. The factors that condition the responses in each country will be identified, emphasizing the global context and international relations, the political-institutional context and the structural inequalities existing in these countries.

## 2. Materials and methods

This protocol focuses on a scoping review of the literature of governance and health surveillance systems responses to COVID-19 in BRICS countries. The method was selected for its capacity to explore specific aspects and gaps surrounding the topic of interest [34], as well as to map study types and related evidence without distinguishing studies based on methodological design [35]. The study used the PRISMA-P reporting guidelines (Preferred Reporting Items for Systematic Review and Meta-Analysis Protocols) [36]. The Joanna Briggs Institute (JBI), a leading institution in developing standards of regulation and methodological standardization for literature reviews, applies five methodological steps to scoping reviews: (1) identification of the research question; (2) identification of relevant previous studies; (3) definition of study eligibility criteria; (4) systematic data retrieval; and (5) summarization of results [37].

This protocol was registered on Open Science Framework (OSF) [https://doi.org/10.17605/OSF.IO/SN5ZY] and Figshare [https://doi.org/10.6084/m9.figshare.25908340] platforms, as recommended by the JBI to ensure the internationalization of research initiatives and methodological transparency throughout all stages of data collection and reporting results.

Also following recommendations, an initial search was carried out to verify the existence of protocols and to register existing research titles that might have had the same aim as this research. The search was conducted in the Open Science Framework and Figshare platforms and the JBI PROSPERO protocol register: no results were found for scientific productions in the theoretical and methodological scope of this research. An initial search for publications within the proposed scope was also carried out on the Scielo, Lilacs, Google Scholar and Researchgate databases; no results were found that directly aligned with the study’s scope.

Based on this initial search, it was considered that the factors of innovation and originality are present in the hypothesis, research question, goals and methodological design proposed in this protocol. The proposed study does not require ethical approval.

### 2.1. Identification of the research question

Governance and responses of health and surveillance systems to COVID-19 in the context of socio-economic inequalities in the BRICS countries are the focus of this systematic review. The focus, question, and hypothesis of this work are detailed in Table 1.

**Table 1:** Focus, question and hypothesis of the scoping review protocol.

Focus	Governance and responses of health and surveillance systems to COVID-19 in the context of inequalities.
**Question**	**General question:** What are the characteristics of the governance and responses of health and surveillance systems to COVID-19 in the BRICS countries, in the face of socio-economic inequalities? **Specific questions:** 1. What political-institutional, operational and communicational governance actions have been implemented by the BRICS countries? To what extent have they contributed to the success of national responses? Have governance actions helped reduce inequality in these countries? 2. How have BRICS health systems been organized to respond to COVID-19? What primary and hospital care actions have been implemented? Have they helped reduce inequality in these countries? 3. What public health surveillance measures have been implemented in the BRICS countries in the face of COVID-19? How have testing and vaccination strategies been organized? Have health information systems been expanded and updated from a digital perspective? Have these measures helped to reduce inequality in these countries?
**Hypothesis**	Structural inequalities, health system configurations, and the political-institutional context can influence the capacity to respond to COVID-19 in the BRICS countries. The effectiveness of the response will depend on the characteristics of government action in the social, economic, political, and health contexts in each country.

Source: the authors.

The construction of the research question was based on the PCC mnemonic, where P represents the population, C the concept, and C the context. The ‘population’ refers to BRICS countries. According to the objectives of this protocol, the guiding concepts are Governance and national coordination of the response to COVID-19; Health system response to COVID-19; Public health surveillance measures in response to COVID-19. The definition and scope of each of these concepts are detailed in Table 2. Regarding the context, the inequalities present in these countries stand out as a key axe of analysis, as they affect the national response capacity and the effects of the pandemic on the population.

**Table 2:** Population, concepts and context of the scoping review protocol.

Population (P)	BRICS countries - Brazil, Russia, India, China, and South Africa.
**Concepts (C)**	**National governance and coordination of the response to COVID-19:** national coordination strategies and intersectoral actions in at least three dimensions: 1) Political-institutional governance, considering the articulation between different sectors (Health, Welfare, Social Assistance, Labor, Economy, among others) and levels of government; 2) Operational governance, dealing with the relationship between public health departments, institutions and services of the health system; and 3) Communicational governance, involving information and engagement actions with civil society, workers’ organizations and companies [5,24]. **Health system response to COVID-19:** set of actions aimed at preventing, diagnosing, and treating COVID-19 to reduce mortality, morbidity, and long-term consequences [12]. They involve the provision of primary and hospital care and services for COVID-19 through national health systems. They may include regulating the operation of these facilities, managing the workforce, and allocating financial resources. **Public health surveillance measures against COVID-19:** Measures aimed at reducing and controlling the incidence of COVID-19 to protect people, especially those in situations of greater vulnerability, and alleviate the pressure on the health system [12]. It involves systematic data collection, production, and dissemination of health information, testing, case monitoring, and vaccination. It can include measures to regulate social distancing and investment in expanding early warning systems, laboratory surveillance, and digital systems.
**Context (C)**	Social inequalities in the COVID-19 pandemic during the period of 2020 to 2024.

Source: the authors.

### 2.2. Identification of relevant studies

#### 2.2.1. Data sources.

The search for scientific articles will be conducted in six databases: BVS Regional Portal, Pubmed, Web of Science, Scopus, Embase, and Dimensions. In addition, references relevant to the topic will be manually selected from the bibliographical references list of the articles found, as well as selected official documents from the World Health Organization (WHO), the Organization for Economic Cooperation and Development (OECD), the Economic Commission for Latin America (ECLAC), the official websites of the countries studied, and the open platforms Our World in Data (OWD), and PoliMap.

#### 2.2.2. Search strategy.

The review will be based on a primary search strategy that will be adapted for each database according to the defined eligibility criteria. The search strategy was constructed of keywords and descriptors selected from the Health Sciences Descriptors (DeCS), in Portuguese and English, and the Medical Subject Headings terms (Mesh). The search strategy was defined after a preliminary bibliographic search that allowed the research object and eligibility criteria to be refined. To ensure comprehensive coverage of the research question, the search strategy was divided into three axes, corresponding to the concepts that guide this protocol: Governance and national coordination during the COVID-19 pandemic; Health system responses to COVID-19; Public health surveillance actions to COVID-19 in the BRICS countries.

The search terms were organized into lines using Boolean operators, to intersect them with terms related to COVID-19 and the BRICS countries. In preliminary searches, however, it was observed that the insertion of terms related to inequalities caused significant reduction in relevant results for the envisioned discussion. As a result, the line on inequalities was only used in the third axis, where the number of results was very significant, and the addition of this line promoted better findings. In addition, country (Brazil, Russia, India, China, South Africa) and date (2020-2024) filters were used in each of the databases. The database-specific search strategies are detailed in Table 3.

**Table 3:** Main search strategy according to study concept.

Study concepts	Search strategy
**National governance and coordination of the response to COVID-19**	(“Health governance” OR Governance OR “National response” OR “Intersectoral collaboration” OR “Intersectoral cooperation” OR “Social participation” OR “Community participation” OR “Social engagement” OR “Communication systems”) AND (COVID-19 OR Pandemic) AND (Brics OR Brasil OR Brazil OR Russia OR China OR India OR “África do Sul” OR “South Africa”)
**Health system response to COVID-19**	(“National response” OR “Delivery of Health Care” OR “Primary health care” OR “Hospital care”) AND (COVID-19 OR Pandemic) AND (Brics OR Brasil OR Brazil OR Russia OR India OR China OR “África do Sul” OR “South Africa”)
**Public health surveillance measures against COVID-19**	(“Health surveillance” OR “Public health surveillance” OR “Health Care Coordination” OR “Monitoring health care” OR “Health information systems” OR “Information systems” OR Vaccination OR Immunization OR Testing) AND (COVID-19 OR Pandemic) AND (BRICS OR Brasil OR Brazil OR Russia OR China OR “África do Sul” OR “South Africa” OR India) AND (“Economic and Social Factors” OR “Factor, Socioeconomic” OR “Factors, Socioeconomic” OR “High Income Population” OR “High-Income Population” OR “High-Income Populations” OR “Inequalities, Social” OR “Inequality, Social” OR “Population, High-Income” OR “Populations, High-Income” OR “Social and Economic Factors” OR “Social Gradient in Health” OR “Social Inequalities” OR “Social Inequality” OR “Social Inequity” OR “Socioeconomic Characteristics” OR “Health inequities” OR “Health Inequalities” OR “Health Inequity” OR “Inequities, Health” OR “Inequity, Health” OR “Health disparities”)

Source: the authors.

### 2.3. Definition of the eligibility criteria

The studies included in the scoping review will be selected by a team of six researchers, divided into three pairs, each assigned to one of the three thematic axes. Each researcher will work independently, according to the PRISMA guidelines, and will compare results at each stage of database refinement. Disagreements will be resolved within each pair or with the help of a third reviewer. At each stage, results will be justified based on the predetermined eligibility criteria outlined below.

For the selection of studies, the criteria include secondary sources in the form of scientific publications such as articles, book chapters, dissertations or theses, available in Portuguese, English, or Spanish.Additional sources will be added manually from national government and institutional documents, as well as official documents from the World Health Organization (WHO), the Organization for Economic Cooperation and Development (OECD), the Economic Commission for Latin America (ECLAC), the official websites of the countries studied, and the Our World in Data (OWD), and PoliMap open data search platforms. The selected works should deal with the period from 2020 to 2024, regarding the responses of the BRICS countries to the SARS-CoV-2 pandemic and its impact on socio-economic inequalities.

Publications such as editorials, introductions and prefaces to works, annals of scientific events, clinical trials, incomplete records, or those that do not answer the research questions based on the concepts and contexts defined will be excluded during the refinement stages. Thus, publications that report the response at a local or regional level without considering elements at a national level, publications that use the time frame but do not address COVID-19, and publications that address COVID-19 from a clinical perspective (treatments, effects of possible therapies, consequences and post-COVID-19 symptoms) will be excluded. Publications that deal exclusively with the diagnosis of COVID-19 in comorbidity with other diagnoses will also be disregarded.

### 2.4. Data retrieval

The data will be retrieved and uploaded to the Rayyan web-tool for the selection process. Three different interfaces will be created following the main axes - Governance and national coordination of the response to COVID-19, Health system responses to COVID-19, Public health surveillance measures in the face of COVID-19 - on which each pair of researchers will work.

The results selected as the final study sample will be organized in a data extraction form, using items presented in Table 4. Additional items may be incorporated into the extraction form as the process evolves.

The data extraction form will undergo a pilot test to identify needs for adaptation to the review’s objectives and questions, possible redundancies, and inaccuracies. The selected material will be gathered in external folders outside the web-tool. The entire process will be registered in the revision report.

### 2.5. Data summarization and reporting

After collecting the data, a descriptive analysis of the selected studies will be conducted to summarize them according to three main axes, distinguishing responses by country and international collaboration among BRICS members.

The results will then be summarized in two reports: (1) Quantitative synthesis, the results will be summarized quantitatively based on the items presented in Table 4; and (2) Thematic analysis, the results will be summarized qualitatively based on Thematic data items (presented in Table 4), aligning with the proposed objectives and hypotheses. The analysis presented in these reports will be widely communicated in accordance with the dissemination plan, outlined in the following section.

**Table 4:** Preliminary items of the data extraction form.

Item	Description
**Title**	Title of manuscript.
**Authorship**	Relation of authors.
**Year of publication**	Year.
**Journal/publisher**	Title of journal or publisher.
**Type of publication**	Article, book chapter, dissertation, thesis, or government document.
**DOI**	DOI of manuscript.
**Language**	English, Portuguese, or Spanish.
**Abstract**	Abstract of manuscript.
**BRICS country**	Brazil, China, India, Russia, South Africa, or comparative studies (if the manuscript involves more than one country).
**Type of evidence source**	Original research (original peer-reviewed articles presenting results from studies using primary and secondary data); Review, Essay, Opinion and perspective article, …
**Aims, purposes, arguments and problem**	Aims, purposes, arguments, and problem developed in the manuscript.
**Study design**	Qualitative studies, Quantitative studies, Mixed and/or multi-method studies, …
**Methodological approach**	Political analyses, Analysis of public health policy, …
**Thematic data** **(to guide basic qualitative data analysis)**	In accordance with the concepts of this scoping review protocol, thematic data will be identified: 1. On the axis Governance and national coordination of the response to COVID-19, the main themes will be: a) Political-institutional governance, b) Operational governance, c) Communicational governance. The secondary themes could be: • Intersectoral coordination and leadership, • Coordination between levels of government, • Relationship between public health departments, institutions and health system services, • Cases of social engagement and community organization, • Communication and information actions (State and society articulation), • Society’s actions to address inequalities, • State actions with an impact on inequalities, • National response [indicate country], • Successful subnational response (related to the national case). 2. On the axis Health system responses to COVID-19, the main themes will be: a) Primary health care, b) Hospital care, c) Implications of inequalities on COVID-19 outcomes. The secondary themes could be: • Regulation of the functioning of health services, • Workforce management, • Allocation of financial resources, • Expansion of the provision of health services, • National response [indicate country], • Successful subnational response (related to the national case), • Society’s actions to address inequalities, • State actions with an impact on inequalities. 3. On the axis Public health surveillance measures in the face of COVID-19, the main themes will be: a) Measures to reduce and control the incidence of COVID-19 to protect people, b) Measures to protect people in situations of greater vulnerability, c) Measures to reduce pressure on the healthcare system. The secondary themes could be: • Systematic data collection, production and dissemination of health information, • Testing and monitoring cases, • Vaccination, • Regulation of social distancing, • Early warning systems, • Laboratory surveillance, • Digitalization of healthcare and digital systems for COVID-19, • National response [indicate country], • Successful subnational response (related to the national case), • Society’s actions to address inequalities, • State actions with an impact on inequalities.

Source: the authors.

## 3. Discussion

The scoping review protocol focuses on the governance and response of health systems to the COVID-19 pandemic in the BRICS countries and represents an original study with high potential to generate innovative and applicable knowledge to strengthen preparedness and responses to health emergencies in these five very important countries in the global south. This protocol was constructed with careful methodological rigor to achieve this objective; however, some limitations must be acknowledged. The search for studies in Portuguese and English may lead to the exclusion of relevant research written in the native languages of the countries under analysis. Additionally, given the recent nature of the pandemic, not all conducted studies have been fully published, which may also result in the omission of pertinent works. Finally, despite well-defined and comprehensive selection criteria, there remains the possibility that certain studies may not be included.

The dissemination plan includes the presentation of results in formats tailored for effective communication with each target audience, including the general public, academics working in the field, and interested international stakeholders. The results will be shared through publications in English and Portuguese, as well as at scientific events dedicated to international cooperation between the BRICS countries in the form of oral presentations. This strategy aims to strengthen discussions that could enhance a comparative analysis of the health systems in these diverse countries - differing in culture, language, politics, economy, geography, and welfare - which are brought together by a political alliance for economic cooperation and development. The recent evolution of BRICS members demonstrates the ever-growing relevance of this group and the importance of deepening knowledge of each country’s health culture.

## Supporting information

S1 Checklist PRISMA-P 2015 checklist (completed_PRISMA-P_checklist.docx).
(DOCX)

## References

[1] Mallah SI, Ghorab OK, Al-Salmi S, Abdellatif OS, Tharmaratnam T, Iskandar MA, et al. COVID-19: breaking down a global health crisis. Ann Clin Microbiol Antimicrob. 2021;20(1):35. doi: 10.1186/s12941-021-00438-7 34006330 PMC8129964

[2] Clemente-Suárez VJ, Navarro-Jiménez E, Moreno-Luna L, Saavedra-Serrano MC, Jimenez M, Simón JA, et al. The Impact of the COVID-19 Pandemic on Social, Health, and Economy. Sustainability. 2021;13(11):6314. doi: 10.3390/su13116314

[3] Yoo JY, Dutra SVO, Fanfan D, Sniffen S, Wang H, Siddiqui J, et al. Comparative analysis of COVID-19 guidelines from six countries: a qualitative study on the US, China, South Korea, the UK, Brazil, and Haiti. BMC Public Health. 2020;20(1):1853. doi: 10.1186/s12889-020-09924-7 33272250 PMC7711256

[4] Haldane V, De Foo C, Abdalla SM, Jung A-S, Tan M, Wu S, et al. Health systems resilience in managing the COVID-19 pandemic: lessons from 28 countries. Nat Med. 2021;27(6):964–80. doi: 10.1038/s41591-021-01381-y 34002090

[5] Machado C, Pereira A, Freitas C. As respostas dos países à pandemia em perspectiva comparada: semelhanças, diferenças, condicionantes e lições. In: Machado CV, Pereira AMM, Freitas CM, editors. Políticas e sistemas de saúde em tempos de pandemia: nove países, muitas lições [online]. Rio de Janeiro: Observatório Covid-19 Fiocruz; Editora Fiocruz;. 2022;323–42. doi: 10.7476/9786557081594.0012

[6] Assefa Y, Gilks CF, Reid S, van de Pas R, Gete DG, Van Damme W. Analysis of the COVID-19 pandemic: lessons towards a more effective response to public health emergencies. Global Health. 2022;18(1):10. doi: 10.1186/s12992-022-00805-9 35120537 PMC8815718

[7] McKee M, Marten R, Balabanova D, Watt N, Huang Y, Finch AP, et al. BRICS’ role in global health and the promotion of universal health coverage: the debate continues. Bull World Health Organ. 2014;92(6):452–3. doi: 10.2471/BLT.13.132563 24940020 PMC4047805

[8] Romaniuk P, Poznańska A, Brukało K, Holecki T. Health System Outcomes in BRICS Countries and Their Association With the Economic Context. Front Public Health. 2020;8 80. doi: 10.3389/fpubh.2020.00080 32296671 PMC7136407

[9] Ebrahim SH, Gozzer E, Ahmed Y, Imtiaz R, Ditekemena J, Rahman NMM, et al. COVID-19 in the least developed, fragile, and conflict-affected countries - How can the most vulnerable be protected?. Int J Infect Dis. 2021;102:381–8. doi: 10.1016/j.ijid.2020.10.055 33130196 PMC7833301

[10] Horton R. The COVID-19 catastrophe: what’s gone wrong and how to stop it happening again. 2nd ed. Cambridge: Polity Press; 2021.

[11] World Health Organization (WHO). Independent Panel for Pandemic Preparedness and Response. COVID-19: make it the last pandemic. Geneva: WHO; 2021. (Cited 2025 Feb 03). Available from: https://theindependentpanel.org/wp-content/uploads/2021/05/COVID-19-Make-it-the-Last-Pandemic_final.pdf

[12] World Health Organization (WHO). Strategic preparedness, readiness and response plan to end the global COVID-19 emergency in 2022. Geneva: WHO; 2022. (Cited 2025 Feb 03). Available from: https://www.who.int/publications/i/item/WHO-WHE-SPP-2022.1

[13] Albuquerque MV de, Ribeiro LHL. Inequality, geographic situation, and meanings of action in the COVID-19 pandemic in Brazil. Cad Saude Publica. 2021;36(12):e00208720. doi: 10.1590/0102-311X00208720 33440421

[14] Sharma V, Scott J, Kelly J, VanRooyen MJ. Prioritizing vulnerable populations and women on the frontlines: COVID-19 in humanitarian contexts. Int J Equity Health. 2020;19(1):66. doi: 10.1186/s12939-020-01186-4 32404178 PMC7218503

[15] Shadmi E, Chen Y, Dourado I, Faran-Perach I, Furler J, Hangoma P, et al. Health equity and COVID-19: global perspectives. Int J Equity Health. 2020;19(1):104. doi: 10.1186/s12939-020-01218-z 32586388 PMC7316580

[16] Chen T, Wang Y, Hua L. “Pairing assistance”: the effective way to solve the breakdown of health services system caused by COVID-19 pandemic. Int J Equity Health. 2020;19(1):68. doi: 10.1186/s12939-020-01190-8 32414384 PMC7226711

[17] Narain JP, Dawa N, Bhatia R. Health System Response to COVID-19 and Future Pandemics. Journal of Health Management. 2020;22(2):138–45. doi: 10.1177/0972063420935538

[18] Haldane V, Morgan GT. From resilient to transilient health systems: the deep transformation of health systems in response to the COVID-19 pandemic. Health Policy Plan. 2021;36(1):134–5. doi: 10.1093/heapol/czaa169 33319220 PMC7799054

[19] Nuzzo JB, Meyer D, Snyder M, Ravi SJ, Lapascu A, Souleles J, et al. What makes health systems resilient against infectious disease outbreaks and natural hazards? Results from a scoping review. BMC Public Health. 2019;19(1):1310. doi: 10.1186/s12889-019-7707-z 31623594 PMC6798426

[20] Pereira AMM, Machado CV, Veny MB, Juan AMY, Recio SN. Governance and state capacities against COVID-19 in Germany and Spain: national responses and health systems from a comparative perspective. Cien Saude Colet. 2021;26(10):4425–37. doi: 10.1590/1413-812320212610.11312021 34730633

[21] Rajan D, Koch K, Rohrer K, Bajnoczki C, Socha A, Voss M, et al. Governance of the Covid-19 response: a call for more inclusive and transparent decision-making. BMJ Glob Health. 2020;5(5):e002655. doi: 10.1136/bmjgh-2020-002655 32371570 PMC7228498

[22] Gilmore B, Ndejjo R, Tchetchia A, de Claro V, Mago E, Diallo AA, et al. Community engagement for COVID-19 prevention and control: a rapid evidence synthesis. BMJ Glob Health. 2020;5(10):e003188. doi: 10.1136/bmjgh-2020-003188 33051285 PMC7554411

[23] Soma K, Termeer CJAM, Opdam P. Informational governance – A systematic literature review of governance for sustainability in the Information Age. Environmental Science & Policy. 2016;56:89–99. doi: 10.1016/j.envsci.2015.11.006

[24] Pereira AMM. Governança, coordenação e estratégias de enfrentamento da crise gerada pela COVID-19: lições aprendidas dos casos chinês, alemão e espanhol. In: Portela MC, Reis LGC, Lima SML, editors. COVID-19: desafios para a organização e repercussões nos sistemas e serviços de saúde [online]. Rio de Janeiro: Observatório COVID-19 Fiocruz, Editora Fiocruz; 2022. p. 63–80. Portuguese. 10.7476/9786557081587.0003

[25] Bambra C, Riordan R, Ford J, Matthews F. The COVID-19 pandemic and health inequalities. J Epidemiol Community Health. 2020;74(11):964–8. doi: 10.1136/jech-2020-214401 32535550 PMC7298201

[26] Marmot M, Allen J. COVID-19: exposing and amplifying inequalities. J Epidemiol Community Health. 2020;74(9):681–2. doi: 10.1136/jech-2020-214720 32669357 PMC7577092

[27] Our World in Data. COVID-19 Pandemic: data explorer. (Cited 2025 Feb 03). Available from: https://ourworldindata.org

[28] Zhu J, Yan W, Zhu L, Liu J. COVID-19 pandemic in BRICS countries and its association with socio-economic and demographic characteristics, health vulnerability, resources, and policy response. Infect Dis Poverty. 2021;10(1):97. doi: 10.1186/s40249-021-00881-w 34238368 PMC8264992

[29] Garcia AS, Curty R, Aguiar AC, Rezende L, Dantas MV. Os BRICS frente à pandemia da COVID-19: uma análise preliminar sobre políticas comparadas. CI. 2021;17(3):33–46. doi: 10.5752/p.1809-6182.2020v17n3p33-46

[30] Moore C. BRICS and Global Health Diplomacy in the Covid-19 Pandemic: Situating BRICS’ diplomacy within the prevailing global health governance context. Rev bras polít int. 2022;65(2):. doi: 10.1590/0034-7329202200222

[31] Ivins C. Inequality matters: BRICS inequalities fact sheet. Rio de Janeiro: BRICS Policy Center. Brasília: Oxfam International; 2013. (Cited 2025 Feb 03). Available from: https://www.ib-inequality-matters-brics-140313-en.pdf;jsessionid=04CD1CE669CED55E589D610F101304B0

[32] Mújica O, Vázquez E, Duarte E, Cortez-Escalante J, Molina J, Silva J. Socioeconomic inequalities and mortality trends in BRICS, 1990–2010. Bull World Health Organ. 2014;92(6):405–12. PMCID: PMC4047800 24940014 10.2471/BLT.13.127977 PMC4047800

[33] Anikin V, Tikhonova N. Poverty and Inequality in BRICS Countries. Sociological Research. 2016;55(5):305–41. doi: 10.1080/10610154.2016.1294432

[34] Munn Z, Peters MDJ, Stern C, Tufanaru C, McArthur A, Aromataris E. Systematic review or scoping review? Guidance for authors when choosing between a systematic or scoping review approach. BMC Med Res Methodol. 2018;18(1):143. doi: 10.1186/s12874-018-0611-x 30453902 PMC6245623

[35] Sucharew H, Macaluso M. Progress Notes: Methods for Research Evidence Synthesis: The Scoping Review Approach. J Hosp Med. 2019;14(7):416–8. doi: 10.12788/jhm.3248 31251164

[36] Moher D, Shamseer L, Clarke M, Ghersi D, Liberati A, Petticrew M, et al. Preferred reporting items for systematic review and meta-analysis protocols (PRISMA-P) 2015 statement. Syst Rev. 2015;4(1):1. doi: 10.1186/2046-4053-4-1 25554246 PMC4320440

[37] Peters MDJ, Godfrey C, McInerney P, Munn Z, Tricco AC, Khalil H. Scoping Reviews (2020). Aromataris E, Lockwood C, Porritt K, Pilla B, Jordan Z, editors. JBI Manual for Evidence Synthesis. JBI; 2024. doi: 10.46658/JBIMES-24-09

